# The 16S rDNA high-throughput sequencing correlation analysis of milk and gut microbial communities in mastitis Holstein cows

**DOI:** 10.1186/s12866-023-02925-7

**Published:** 2023-07-07

**Authors:** Chenxi Jiang, Xiaolu Hou, Xiaona Gao, Pei Liu, Xiaoquan Guo, Guoliang Hu, Qingqing Li, Cheng Huang, Guyue Li, Weile Fang, Wanrui Mai, Cong Wu, Zheng Xu, Ping Liu

**Affiliations:** 1 Jiangxi Provincial Key Laboratory for Animal Health, Institute of Animal Population Health, College of Animal Science and Technology, Jiangxi Agricultural University, Nanchang, 330045 People’s Republic of China; 2 Guangxi Vocational University of Agriculture, Nanning, 530007 Guangxi China; 3 Department of Mathematics and Statistics, Wright State University, Dayton, OH 45435 USA

**Keywords:** Holstein cow, Mastitis, Milk microorganism, Gut microbial community, 16S rDNA high-throughput sequencing

## Abstract

This study aimed to understand the changes in the milk and gut microbiota of dairy cows with mastitis, and to further explore the relationship between mastitis and the microbiota. In this study, we extracted microbial DNA from healthy and mastitis cows and performed high-throughput sequencing using the Illumina NovaSeq sequencing platform. OTU clustering was performed to analyze complexity, multi-sample comparisons, differences in community structure between groups, and differential analysis of species composition and abundance. The results showed that there were differences in microbial diversity and community composition in the milk and feces of normal and mastitis cows, where the diversity of microbiota decreased and species abundance increased in the mastitis group. There was a significant difference in the flora composition of the two groups of samples (*P* < *0.05*), especially at the genus level, the difference in the milk samples was *Sphingomonas* (*P* < 0.05) and *Stenotrophomonas* (*P* < 0.05), the differences in stool samples were *Alistipes* (*P* < 0.05), *Flavonifractor* (*P* < 0.05), *Agathobacter* (*P* < 0.05) and *Pygmaiobacter* (*P* < 0.05). In conclusion, the microbiota of the udder and intestinal tissues of dairy cows suffering from mastitis will change significantly. This suggests that the development of mastitis is related to the endogenous pathway of microbial intestinal mammary glands, but the mechanisms involved need further study.

## Introduction

Mastitis is an inflammatory reaction in mammary tissue caused by microbial invasion and infection as well as physical or chemical damage, which can affect the milk quality and performance of cows and cause serious economic losses in dairy farming [1, 2]. As an organ linked with the outside environment, the microbiota in the mammary gland is closely associated with mammary fitness [3]. For a long time, the main pathogenic factor of mastitis is believed to be the invasion of pathogenic bacteria from the outside environment and infection of the breast [4]. Mastitis can occur when opportunistic pathogens invade and colonize the mammary gland, however, recent studies have shown that the microorganisms that cause mammary gland infection have an endogenous pathway, gastrointestinal flora also plays an important role in regulating mastitis [5]. When the normal microbial community in the mammary gland is destroyed, the probability of pathogen infection will increase, resulting in breast inflammation [6].

The intestinal flora is a diverse and numerous group of microorganisms that are involved in the gastrointestinal tract participates in nutrient digestion and absorption, specific immune response, and defense of pathogen colonization. Conversely, gut microbiota dysbiosis may be triggered in systemic inflammatory diseases in the host, which can cause intestinal diseases, liver diseases, high blood pressure and immune diseases of the body [7, 8]. Numerous studies have shown that dysbiosis in the gut microbiota is associated with liver disease, liver damage can cause altered gut microbiota and bacterial overgrowth [9]. In addition, gut microbiota is associated with the development of pulmonary hypertension, which lead to changes in the composition of the gut flora [10]. Previous studies have demonstrated that changes in the intestinal microbiota can lead to the proliferation of specific pathogenic bacteria that can subsequently enter the breast via the gut-mammary pathway, leading to the development of mastitis [11]. It is reported that researchers transplanted the feces of mastitis cows into sterile mice and found that the mice showed mastitis symptoms and caused inflammation in the serum ( IFN-γ, IL-17, and endotoxin) and colon (increased IL-1β), which did not occur when transplanting healthy cows feces [12]. This evidence indicated that the gut microbiota is associated with the development of mastitis in dairy cows. In recent years, studies have shown that gut microbial homeostasis is closely related to the development of mastitis in dairy cows and that disturbances in the gut microbiota can lead to the development of mastitis. However, most studies have been conducted in humans and mice, and fewer studies have been conducted in dairy cows [12]. Therefore, this study was conducted to investigate the relationship between mastitis and intestinal microorganisms by analyzing the milk and intestinal flora of cows with mastitis.

In this study, milk and rectal stool samples were collected to investigate the link between mastitis disease and intestinal milk. The microflora in milk can be somewhat representative of the microbial profile of the entire breast tissue. Fecal samples are easy to collect, and as the end product of the metabolic system of the digestive tract, their microorganisms are closely related to those in the gastrointestinal tract and can be used as a representative of the microorganisms in the gastrointestinal tract. We obtained milk and faecal samples from Holstein cows from a cattle farm in Guangxi and analyzed the milk and intestinal flora structure of Holstein cows by 16S rDNA high-throughput sequencing technology to explore the relationship between intestinal microbial communities and milk microorganisms, and to analyze the correlation between mastitis and microbial regulation in cows, providing a scientific basis for the diagnosis and prevention research of mastitis in cows.

## Materials and methods

### Animals and sample collection

Cows were selected from a well-managed large-scale dairy farm in in Jiangnan District, Nanning, Guangxi, China. We first performed clinical examination on cows, using SCC (the number of leukocytes per milliliter of fresh milk) as the criterion. The dairy cows were judged to suffer from clinical mastitis based on the obvious symptoms of redness of either udder, milk curdling, discoloration, and when the somatic cell count in milk was more than 5.0 × 10^5^cells/mL [13]. Fresh milk and faecal samples were collected from control and experimental cows in sterile EP tubes, and the numbers were randomly placed in ice boxes for cryopreservation and sent to the laboratory. After initial screening of the samples, we obtained a total of 22 usable samples, with milk and faeces numbers NM1-NM5 and NF1-NF6 from normal cows and MM1-MM5 and MF1-MF6 from cows with mastitis, respectively.

### DNA Extraction, PCR amplification and sequencing

Total microbial DNA was extracted from the samples according to the Genomic DNA Extraction Kit, and the concentration and purity of the DNA samples were determined using agarose gel electrophoresis. The samples were diluted to 1 ng/µL with sterile water. The diluted genomic DNA was used as a template for targeted amplification of the V3-V4 region of the 16S rDNA using specific primers. The product was tested for concentration and purity by 2% agarose gel electrophoresis. The DNA library was built using a library building kit. After the library was quantified and tested by Qubit, it was sequenced using NovaSeq6000, and the sequencing was entrusted to Beijing Novo gene Technology Co.

### Sequencing information analysis

The Uparse software was used to perform chimera removal and clustering analysis on the data, and the number of operational taxonomic units (operational taxonomic units, OTUs) of each sample was obtained through the standard clustering of 97% similarity, and the sequence with the highest frequency was selected as represents a sequence. The representative sequence and SSURef are used as a reference database to annotate the sequence, so as to classify each OTU as a species, and then obtain the OUT according to the number of sequences in each OTU, and use the Venn diagram to visually display the OTU between samples Condition. α-diversity analysis was performed on the OTU results, and the complexity between samples was displayed through β-diversity analysis, and use the weighted UniFrac distance to perform principal coordinate analysis (PCoA) on the sample data to show the diversity differences between samples. Finally, the species abundance is analyzed by comparing with the database, and the species are classified.

### Statistical analyses

Statistical analysis of the experimental data was carried out using SPSS software. Results are presented in the format of mean ± SE. Comparisons between groups were conducted using ANOVA and t-tests. Results with *P* values < 0.05 were regarded to be “significant”and results with *P* values < 0.01 were regarded to be “very significant”.

## Results

### Sample sequence information statistics

The raw microbiome sequencing data obtained on the Illumina NovaSeq platform contain a total of 1,580,497 raw sequences. After filtering and optimization, a total of 1,191,596 valid sequences were obtained. Table 1 shows the number of sequences of samples in the original data, the initial filtered data and the QC filtered data, and we are able to find some differences in the data for each sample.

**Table 1:** Sample sequencing sequence statistics

Sample ID	Raw Data	Clean Data	Effective Data	Ratio	Sample ID	Raw Data	Clean Data	Effective Data	Ratio
MM.1	69,710	66,361	60,232	64.65	MF.3	91,861	88,075	67,323	70.86
MM.2	47,335	45,136	41,343	62.97	MF.4	79,534	76,336	55,887	67.80
MM.3	50,665	48,364	44,822	64.74	MF.5	88,669	84,807	84,807	66.39
MM.4	82,296	77,398	69,508	61.78	MF.6	84,045	79,338	58,034	65.24
MM.5	58,026	54,873	48,950	64.12	NF.1	90,650	86,444	60,664	64.33
NM.1	80,360	75,770	61,351	63.26	NF.2	83,651	78,372	55,769	62.62
NM.2	53,832	50,780	43,882	60.41	NF.3	94,714	90,324	65,421	66.41
NM.4	76,889	74,499	61,577	59.77	NF.4	95,472	91,285	67,102	67.46
MF.1	85,504	81,161	59,163	65.83	NF.5	88,536	84,190	62,193	67.05
MF.2	88,440	84,523	63,718	69.16	NF.6	90,308	85,789	59,850	63.12

Raw data refers to splice sequence date, Clean data is the sequence after filtering low quality and short length, Effective data is the sequence after filtering chimeras into later data analysis

**Fig. 1:**
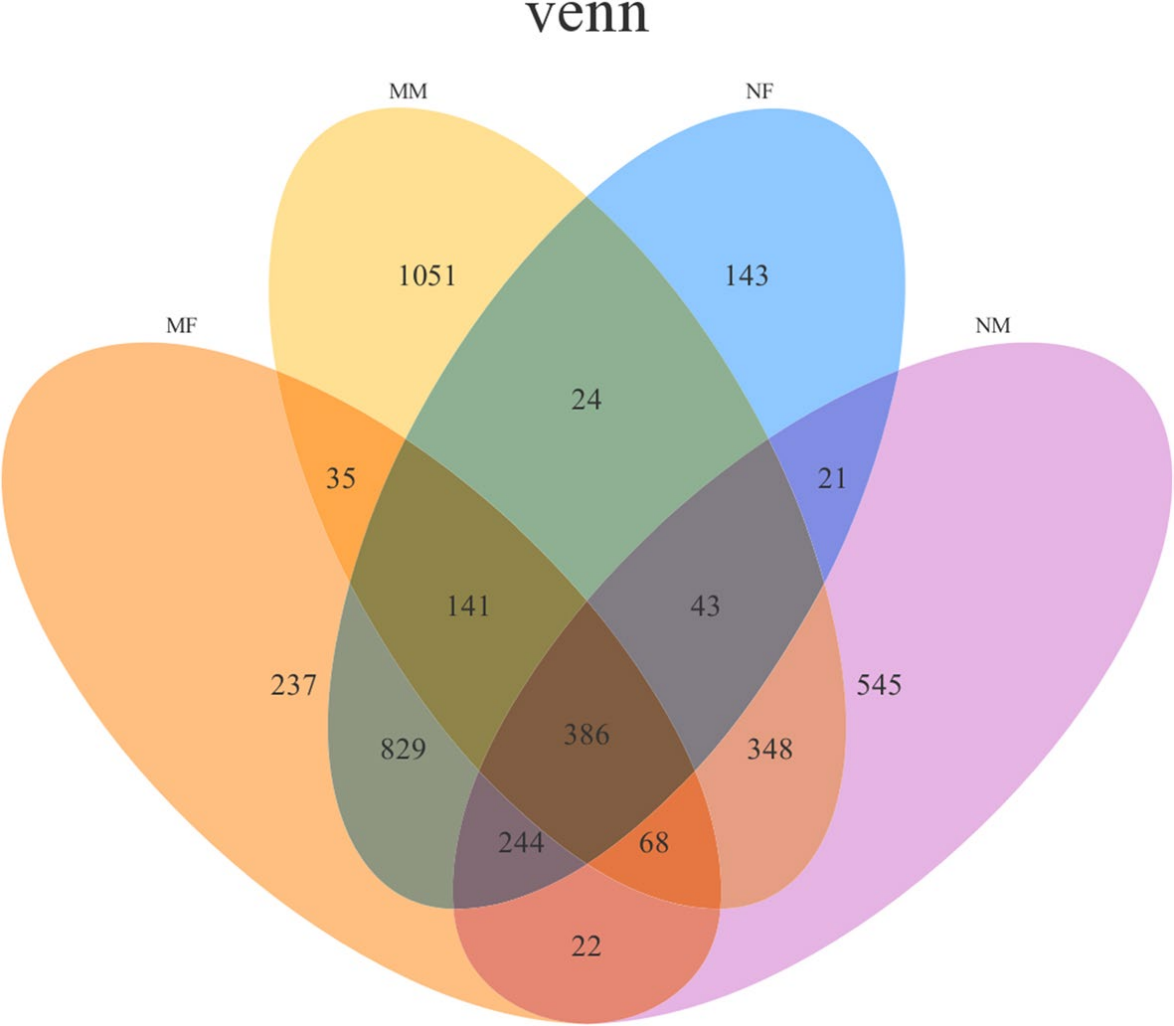
Venn diagram representation of the OTUs shared among different groups

As shown in Fig. 1, the overlap of OTUs in the different samples is visualized. Further analysis of the sequence of samples MM, NM, MF, and NF (2096, 1677, 1962, and 1831 OTUs, respectively) showed that 386 OTUs were detected in all samples, which constituted the core bacteria OTUs. There were 1051(MM), 545(NM), 237 (MF) and 143(NF) OTUs identified by exactly one group, which constituted the group specific independent OTUs, indicating that there are some differences in the bacterial composition of different group.

### Alpha diversity of the microbiota

**Fig. 2:**
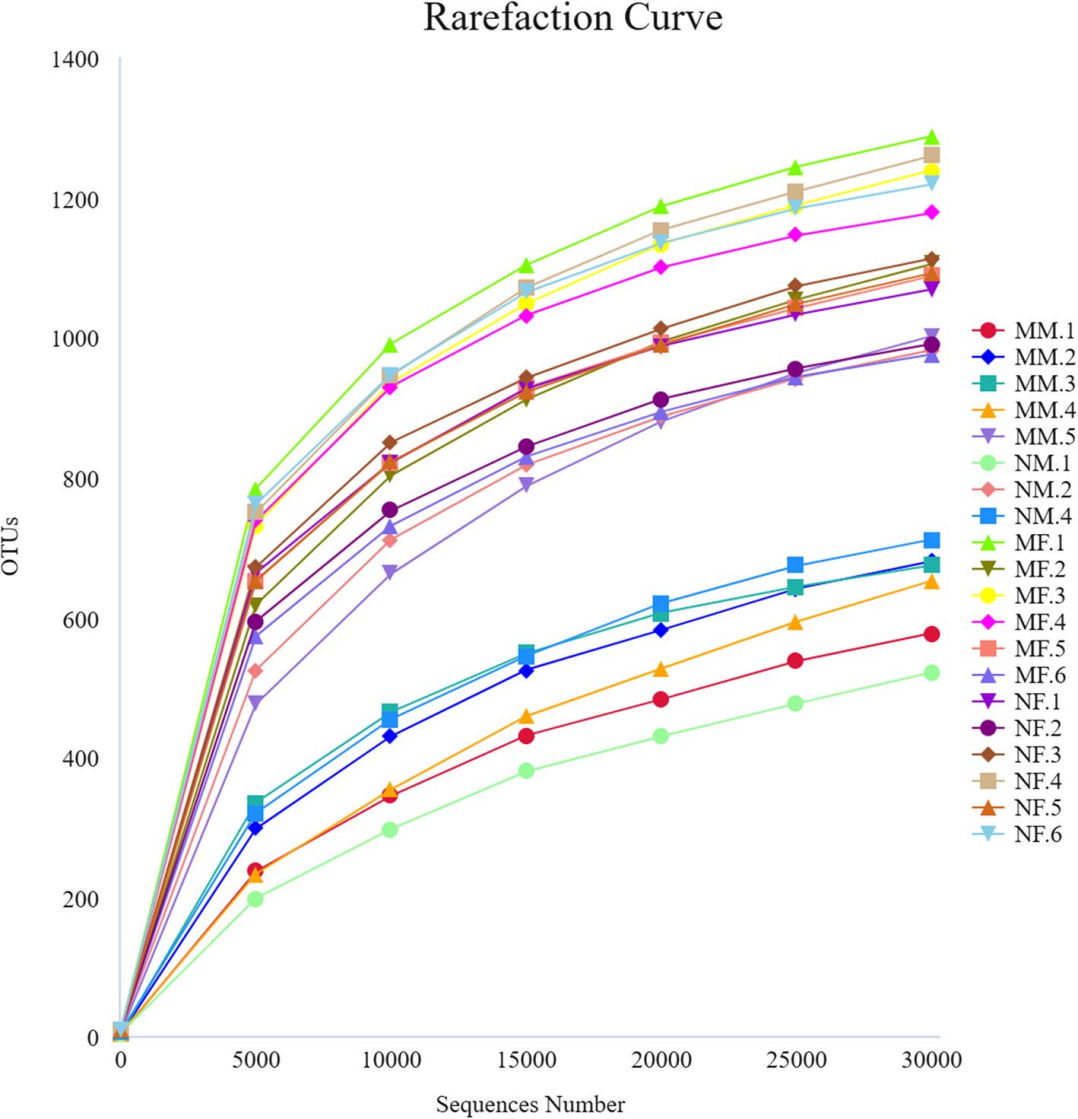
Rarefaction Curve of each sample

Sample complexity analysis can reflect the richness and diversity of the microbial community within the sample. As shown in Fig. 2, it can be seen from the rarefaction curve that when the sequencing depth reaches a certain level, the curve tends to be smooth, and the number of OTUs is close to saturation, which meets the needs for subsequent analysis. The sequencing of this sample is reasonable.

**Fig.3:**
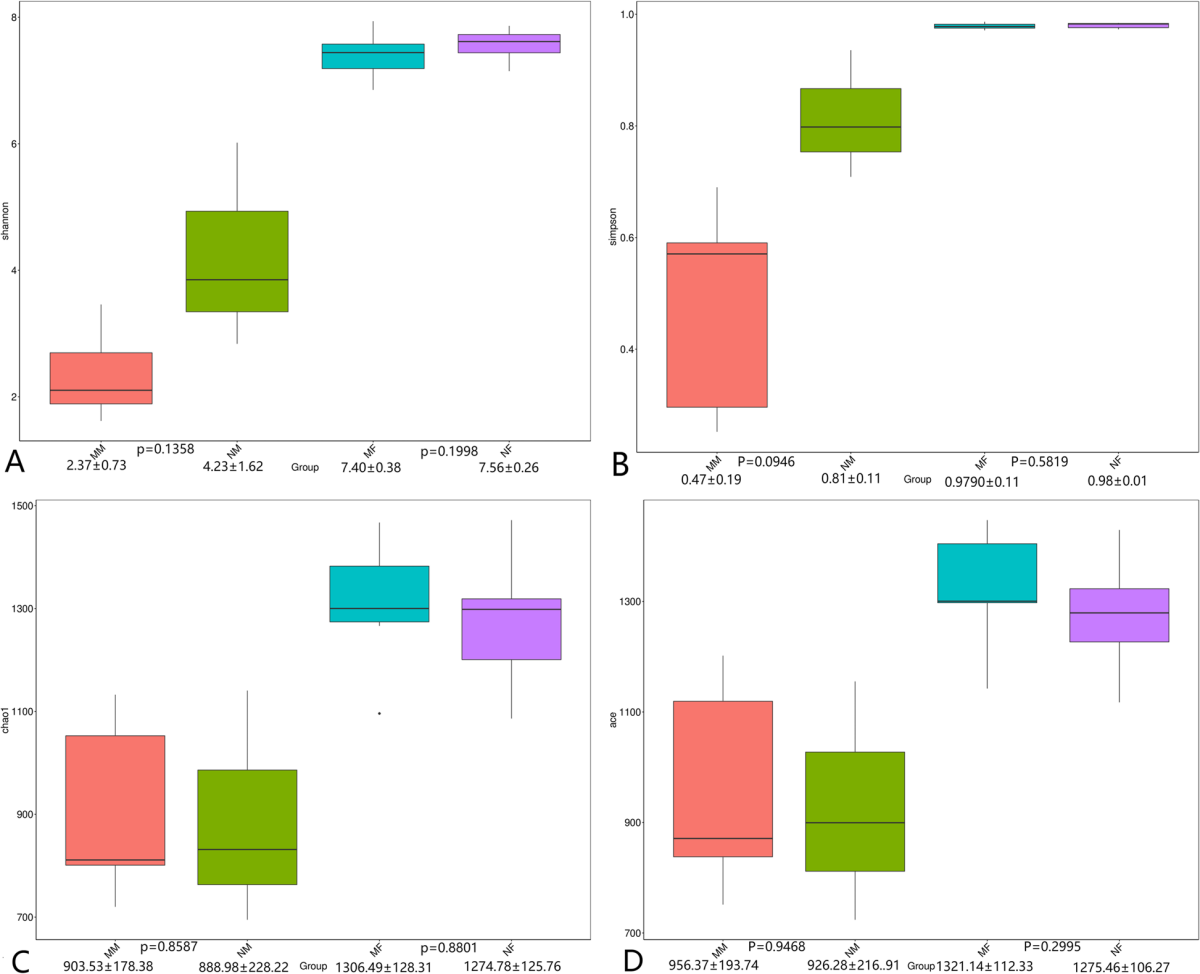
Alpha diversity estimates of the bacterial communities **A**-**D**: Box plots of bacterial alpha diversity assessed by Shannon index, Simpson index, Chao index, and Ace index. Different numbers in the graph indicate different α-diversity indices and *p*-values between groups. The three lines from bottom to top are: first quartile, median and third quartile. Outliers are represented by Outliers are represented by “.”

The microbial diversity and species richness can be measured and indicated by ACE, Chao1, Shannon and Simpson indexes. As shown in Fig. 3 compared with the normal group, the ACE, the Chao1, the Shannon and Simpson indexes all indicated that there was a decreased trend in the species diversity and the species abundance increased in the MM group and MF group, but the difference was not significant (*P* > *0.05*).

### Beta diversity of the microbiota

**Fig. 4:**
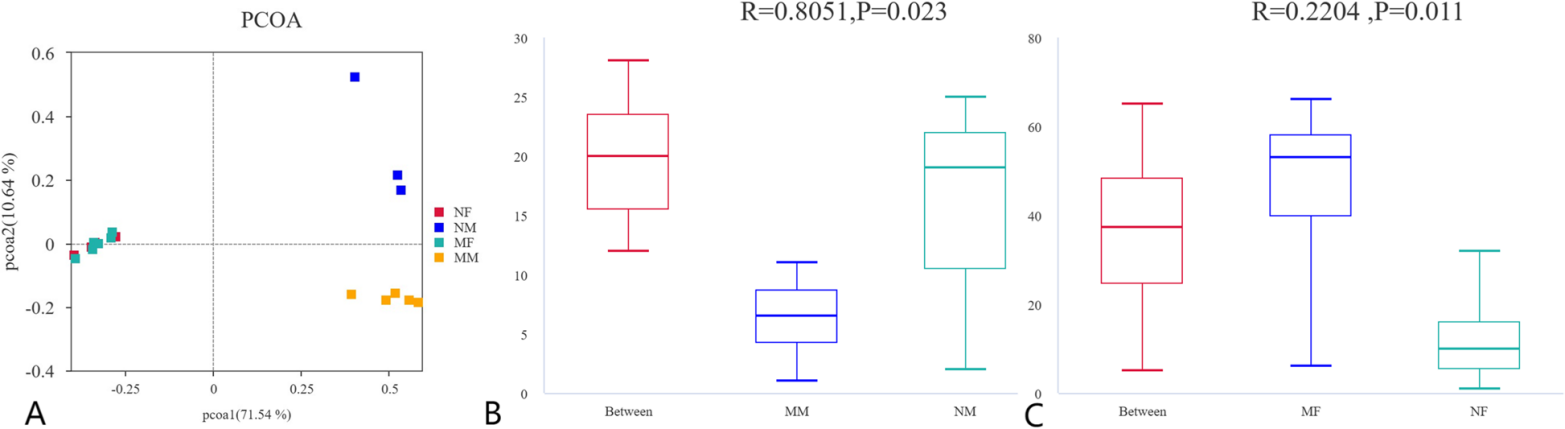
Analysis of differences between microbial groups in samples **A** Principal coordinate analysis (PCoA). **B** Anosim analysis of milk samples. **C** Anosim analysis of faecalis samples. The R-value and *P*-value in the graph represent the confidence level of the sample statistics, where R-value is between (-1, 1) and R-value is greater than 0, indicating a significant difference between groups; *P* < 0.05 indicates that the statistics are significant

We performed principal coordinate analysis (PCoA) on all milk and fecal samples to explore differences in the milk and rectal flora of healthy and mastitis cows. As shown in Fig. 4A, the two groups of milk samples showed great differences in the flora, and the sample points were scattered in the upper and lower right areas of the PCoA plot. The two groups of fecal samples had similar microbiota composition, and their sample points were clustered in the left area of the graph. In addition, it can be seen from the figure that the composition of the flora in the milk and rectal stool samples is very different, and the two groups of samples are distributed in the left and right regions of the PCoA plot.

As shown in Fig. 4BC, we performed an Anosim analysis on the sample data with an R value < 0 and *p* < *0.05,* indicating that the differences between the two groups of samples were significant and that the between-group differences in the samples were greater than the within-group differences. This analysis shows that the grouping of the samples was reasonable and that there were differences between the samples, suggesting that the onset and progression of mastitis affects the composition of the microflora of the breast and gut.

### Microbiological taxonomy

**Fig. 5:**
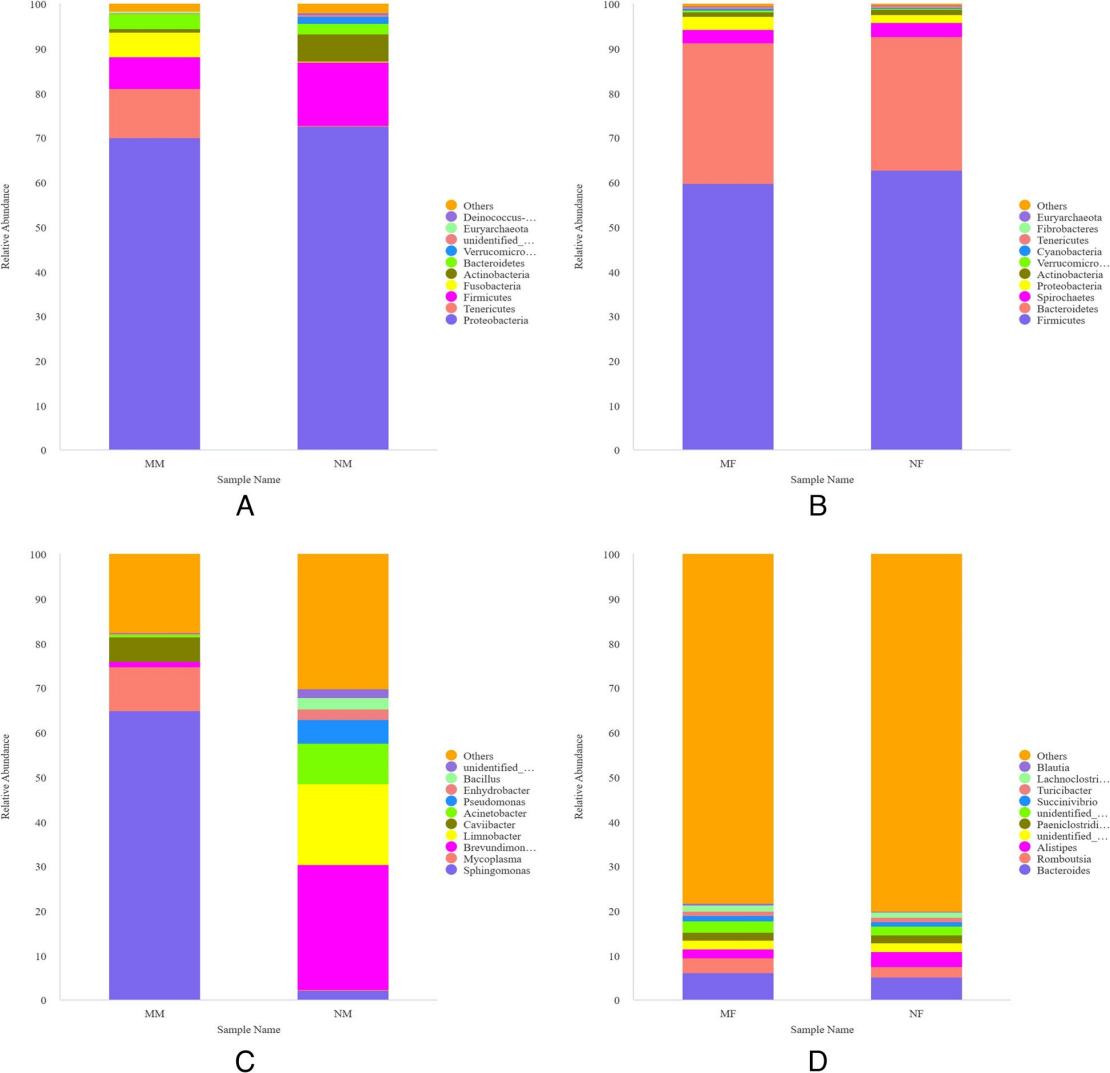
Relative abundances at the phylum level and genus level.  **A**&**C** Mastitis milk and normal milk. **B**&**D** Mastitis fecal and normal fecal

We adopted UPARSE method to conduct OTUs clustering and species annotation analysis on the effective sequences of the samples [14]. Relative abundance column chart was made to represent the composition of the top 10 species with the largest abundance in each sample at the phylum level. As shown in Fig. 5AB, at the phylum level, there were significant differences in the bacterial community structure of the two groups of milk samples. There are 5 phyla with a relative abundance greater than 1%, of which the common dominant phyla are *Proteobacteria, Firmicutes, Bacteroidetes* and *Actinobacteria*. The sum of their abundance accounts for about 87% of the overall abundance, and *Tenericutes* and *Fusobacteria* were present in the mastitis group. The two groups of intestinal fecal samples were relatively stable and similar in structure, with a total of 9 phyla with a relative abundance greater than 0.1%. The dominant bacterial phyla were *Firmicutes*, *Bacteroidetes*, *Spirochaetes*, and *Proteobacteria*, of which the sum of the abundances of *Firmicutes* and *Bacteroidetes* was approximately 90% of the overall abundance. By comparing milk samples and stool samples, we found that milk samples and rectal stool samples were similar in phylum-level bacterial composition.

**Fig. 6:**
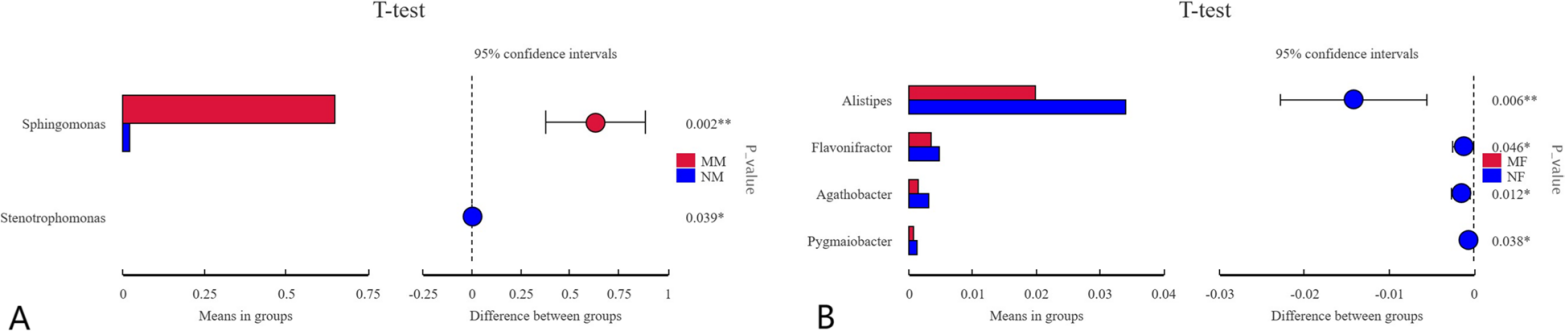
T-test sample group interspecies variance analysis graph. **A** Mastitis milk and normal milk. **B** Mastitis fecal and normal fecal

We also made relative abundance plots at the genus level. As shown in Fig. 5CD, at the genus level, there are significant differences between the two groups of milk samples. The common dominant bacteria genera are *Sphingomonas*, *Brevundimonas* and *Acinetobacter*. Among them, the abundance of *Sphingomonas* in mastitis milk samples increased significantly, while the abundance of *Brevundimonas* and *Acinetobacter* decreased significantly, and *Mycoplasma* and *Caviibacter* appeared. The bacterial composition of rectal fecal samples was similar between the two groups. Compared with the normal group, the abundances of unidentified *Ruminococcaceae*, *Bacteroides*, *Blautia*, *Succinivibrio*, *Paeniclostridium*, *Lachnoclostridiumin* and *Romboutsia* increased in the mastitis group, while the abundances of *Alistipes* and *Turicibacter* decreased. Comparing the two groups of samples, we found that the composition of microorganisms in feces and milk is quite different at the genus level. It can be seen from the above that there were differences in the samples at the genus level, so we performed t-test based on the samples. As shown in Fig. 6, we found the largest difference in microbes in the two groups of milk samples is *Sphingomonas* (*P* < *0.05*), followed by *Stenotrophomona* (*P* < *0.05*). Among the two groups of rectal stool samples, *Alistipes* had the most significant difference in microorganisms (*P* < *0.05*), followed by *Flavonifracto*r (*P* < *0.05*), *Gathobacter* (*P* < *0.05*), and *Pygmaiobacter* (*P* < *0.05*).

**Fig.7:**
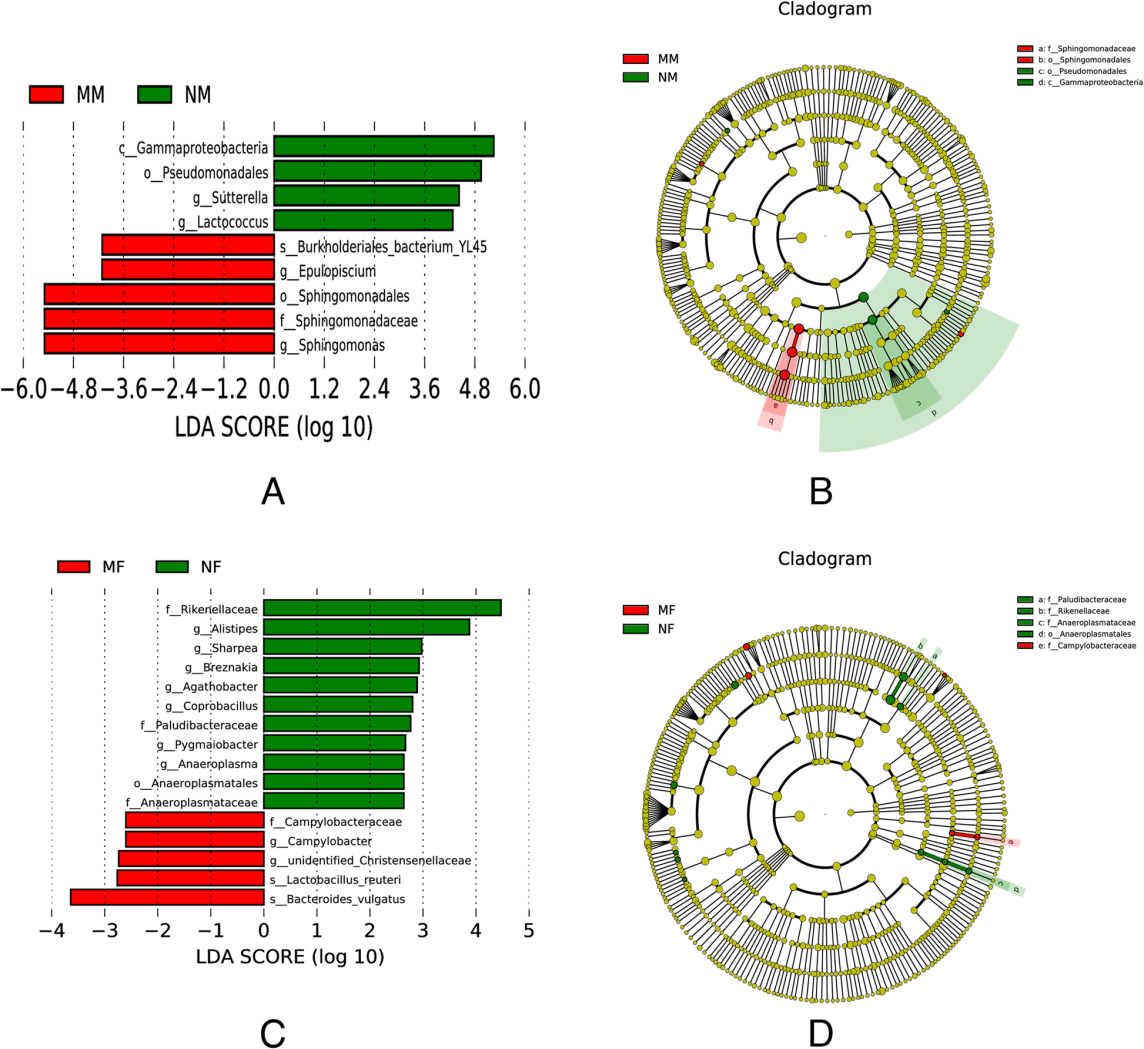
Linear discriminant analysis Effect Size (LEfSe) Analysis of Samples. **A** Histogram of LDA value distribution of milk samples (LD < 4). **B** LEfSe cladogram of milk samples. **C** Histogram of LDA value distribution of rectal stool samples (LD < 4). **D** LEfSe cladogram of rectal stool samples

We also performed Linear discriminant analysis Effect Size (LEfSe) analysis on the samples, which enabled comparisons between multiple groups, and subgroup comparisons within group comparisons to find species with significant differences in abundance between groups. As shown in Fig. 7A, the bacteria that contributed the most to the differences at the genus level between the two groups of milk samples were: *Sutterella*, *Lactpcoccus*, *Epulopiscium*, and *Sphingomonas* at the genus level. In the LEfSe multilevel species-level analysis (Fig. 7B), *Sphingomonadaceae* and *Sphingomonadaceae* were significantly enriched in mastitis cow samples, and *Gammaproteobacteria* and *Pseudomonadales* were significantly enriched in normal cow samples. As shown in Fig. 7C, the bacteria that contributed the most to the differences at the genus level between the two groups of fecal samples were: *Alistipes, Sharpea, Breznakia, Agathobacter, Coprobacillus, Pygmaiobacter, Anaeroplasma, Campylobacter*, and *unidentified-Christensenellaceae*. In the LEfSe multilevel species-level analysis (Fig. 7D), *Anaeroplasmatales, paludibacteraceae, Rikenellaceae*, and *Anaeroplasmataceae* were significantly enriched in normal group cow samples, and.

*Campylobacteraceae* was significantly enriched in mastitis cow samples.

## Discussion

To investigate the relationship between mastitis disease and milk and gut microbiota in dairy cows, we analyzed the microbiota of samples based on 16S rDNA gene sequencing and found significant differences in the microbiota composition of mastitis samples compared with normal samples. Microbial diversity decreased and species abundance increased in the mastitis group compared to the normal group, suggesting that the occurrence of mastitis promotes dysbiosis of the normal microbiota, as evidenced by the accumulation of pathogenic bacteria and depletion of beneficial commensal bacteria [15]. We analyzed the source of milk flora and intestinal flora, and found that there are bacteria from the gastrointestinal tract in milk samples, such as *Sphingomonas, Stenotrophomonas, Sutterella, Lactpcoccus,* and *Epulopiscium*, which indicates that the occurrence of mastitis may be related to the endogenous pathway of the gut microbiota. Studies have shown that cows with mastitis have significantly reduced food intake and exhibit abnormal nutrient metabolism, which is detrimental to the increase of intestinal commensal bacteria, further affecting the amount of beneficial commensal bacteria entering the mammary gland through the endogenous pathways [16]. These all indicate that there is a certain link between the occurrence of mastitis and milk microbes and gut microbes.

In this study, experimental animals were grown under the same environmental and feeding management practices. We collected milk and intestinal fecal samples and found no significant differences in microbial communities among the samples at the phylum level, but the differences at the genus level were very pronounced. We first performed a phylum level analysis and found that the dominant flora common to both groups of milk samples were *Proteobacteria, Firmicutes, Bacteroidetes* and *Actinobacteria*, which accounted for more than 87% of the total bacteria groups. The results are similar to previous studies on milk samples from Holstein cows in clinically healthy areas [17]. Although the predominant flora of the milk samples did not change, the mastitis milk group showed a significant change in the flora abundance, with decreased abundances of *Proteobacteria, Actinobacteria, Verrucomicrobia* and *Firmicutes*, and increased abundances of *Tenericutes, Fusobacteria* and *Bacteroidetes*. This indicated that the normal flora structure of the organism is disrupted, its normal homeostasis was unbalanced, and the rapid change in bacterial abundance led to the occurrence of mastitis. Particular attention was paid to the abundance of *Tenericutes* and *Fusobacterium,* which were more abundant in the mastitis milk group than in the normal group, and both of these phyla contain important bacterial species, such as *Mycoplasma* and *Fusobacterium necrophorum*, which have been associated the etiopathogenesis of CM [18]. We detected these two bacteria with high abundance, indicating that these two bacterial phyla are closely related to the occurrence of mastitis disease. There was no significant difference in the composition of the bacterial flora of the two groups of stool samples at the phylum level, but there were differences in the sample flora at the genus level. The dominant flora in the intestinal tract of the two groups of cows were *Firmicutes* and *Bacteroidetes*, accounting for more than 90% of the total flora, which is consistent with previous reports [19, 20]. There was no significant difference between the two groups of dominant microflora in the intestines of dairy cows (*P* > *0.05*), which may be due to the existence of core microflora in the digestive tract of dairy cows, and its abundance is always stable, generally not changing with the changes in diet, physiological period and environment [21]. We analyzed the source of intestinal flora and found that the bacteria in the samples were mainly gastrointestinal flora, which are involved in the degradation of most nutrients and the generation of a series of metabolites in the gut of dairy cows. They can degrade fiber, hemicellulose, starch, protein and lipids, and produce succinic acid, lactic acid, amino acids, ammonia and fatty acids, which plays an important role in dairy cows' intestinal flora. We analyzed milk samples and found that the ratio of the relative abundance of *Firmicutes* and *Bacteroidetes* was reduced in the mastitis group, and this reduced ratio indicates a reduced nutritional supply and decreased immunity of the organism. This suggests that the occurrence of mastitis disease affects the normal function of the intestinal tract and contributes to changes in the intestinal flora [22, 23].

We then analyzed the samples with t-tests at the genus level and found a significant difference between the two groups. Related studies have shown that the dominant bacteria that play important roles in dairy cows with different mammary gland health status are relatively stable at the phylum level, but there are significant differences at the genus level, which is consistent with our findings [19]. Changes in *Sphingomonas* were significantly different in our cow milk samples. During mastitis in dairy cows, the body will generate an immune response to resist the interference of external unfavorable factors, and *Sphingomonas* from *Proteobacteria* participates in the immune regulation of the body, which can reduce the maturation ability of participating effector CD8^+^T cells (also referred as killer T cells), thus hindering the construction of CD8^+^ anti-tumor cytotoxic T cells [24]. At the same time, studies have found that the most common bacteria in latent mastitis and clinical mastitis is *Sphingomonas*, and there is a strong relationship between the increase in the proportion of this bacteria and the increase in SCC. The abundance of this bacterium was found to be significantly increased in the samples, and we speculate that the occurrence of mastitis has a certain relationship with this genus of bacteria [25]. We also observed differences in samples of *Stenotrophomonas*, a kind of pathogenic bacteria which is strictly aerobic and can infect people and animals. It will cause a decrease in the feed intake and milk yield of dairy cows, with high fever and shortness of breath, accompanied by nervous system symptoms, and even cause multiple organ failure or even death. A significant increase in this bacterium was found in dairy cow mastitis, thus, we speculate that after cows are infected with this pathogenic bacterium, it acts on the mammary gland tissue, causing inflammation of the mammary gland tissue, which leads to the disorder of the flora in the milk, and a large number of *Stenotrophomonas* appear in hyperplasia [26]. The above analysis indicated that significant increases in *Sphingomonas* and *Stenotrophomonas* were associated with mastitis disease. In the gut microbiota samples of mastitis, the abundance of *Alistipes* was significantly reduced. As an important genus in the gut, the bacteria have protective effects on liver fibrosis, cancer immunotherapy and cardiovascular disease, and it has a growth advantage in the intestinal tract of obese patients. We speculate that dairy cows are obese due to excess nutrition during milk production, and this bacterium can have predominant growth in the gut. However, dairy cows suffer from mastitis due to weakened body functions, so the abundance of this genus is significantly reduce [27].

By analyzing the source of bacteria at the genus level of the samples, we found that there are a large number of bacteria from the intestinal flora in the milk samples, such as *Sphingomonas*, *Stenotrophomonas* and *Sutterella*. Among them, *Sphingomonas* and *Stenotrophomonas* are opportunistic pathogens, and we have already analyzed them in the previous analysis. Members of *Sutterella* are important symbiotic bacteria in the intestinal tract, which have slight pro-inflammatory ability in some human diseases, but do not contribute to the epithelial homeostasis destruction related to the imbalance of microbiome and the increase of proteobacteria. They will over-secrete IgA protease and degrade IgA, thus reducing the concentration of IgA in intestinal mucosa and damaging the intestinal antibacterial immune response function. Its abundance is negatively correlated with the levels of inflammatory cytokines (IL-12, IL-13 and IFN-γ) [27–29].We found this genus in milk samples and it decreased in abundance in the mastitis group, indicating that the body was at an inflammatory level with a large increase in inflammatory cells. Studies have shown that during the period of mastitis, the abundance of inflammation-related microorganisms and pro-inflammatory metabolites in the rumen and feces of dairy cows increased significantly, while the abundance of beneficial symbionts decreased drastically [30, 31]. This suggests that our analysis is consistent with the findings that the occurrence of mastitis is associated with changes in the abundance of this bacterium.

Our results show that there is a certain relationship between bovine mastitis and changes in intestinal and milk flora. Moreover, recent studies have shown that the structure of the intestinal microbiota and the pathogenesis of mastitis are closely related, and disturbances in the intestinal microbiota can lead to the development of mastitis [12]. And we have found that mastitis in mice causes disruption of the gut microbiome, increasing the number of pathogenic bacteria and decreasing the number of beneficial bacteria in the gut [32]. Our experimental results show that the intestinal flora of cows with mastitis changes significantly and that there is a correlation between the milk microflora and the intestinal flora. Therefore, we conclude that the occurrence of mastitis may be related to the endogenous pathway of microbial "gut-mammary gland". During cow mastitis, intestinal flora will enter the udder tissue through the endogenous pathway, and bacterial translocation will occur, resulting in the disorder of the flora in the udder tissue, which is manifested by changes in the composition and abundance of the flora. This speculation has been strongly supported in the research of Young et al., but the mechanism needs further study [6, 33]. From this it is clear that the invasion and infection of exogenous pathogenic bacteria may not be the only pathogenic factor of cow mastitis, and gastrointestinal flora may enter the mammary gland through the "gut breast" endogenous pathway, which gastrointestinal flora disorder will also cause cow mastitis. In addition, from our experimental results, we found that there were significant differences between mastitis and normal intestinal flora in cows, indicating that the occurrence of mastitis and milk intestinal flora have some connection. Current research on mammary gland-related diseases (such as mastitis and breast cancer) has focused on humans and mice, and reports on mastitis in dairy cows are extremely limited. Therefore, future studies need to further elucidate the relationship between the onset of mastitis and the gastrointestinal tract in dairy cows, further analyze the correlation between gut microbes and the disease, and provide references for the prevention and control of mastitis disease in dairy cows.

## Conclusion

In addition to the invasion and infection of exogenous pathogenic bacteria, the disorder of rumen and intestinal flora can also cause mastitis. In this study, we used 16S rDNA gene amplicon sequencing technology to analyze the diversity of bacterial flora in milk and feces of Holstein dairy cows, and found that the composition of the bacterial flora was significantly changed due to the influence of mastitis in cows. During the occurrence of mastitis in dairy cows, the composition of breast microbiome and intestinal microbiome in dairy cows will change, and the flora abundance will change. This suggests that the development of mastitis is related to the endogenous pathway of microbial intestinal mammary glands, but the mechanisms involved need further study.

## Data Availability

The complete data set was submitted to the National Biotechnology Information Center (NCBI) short-reading archival database with the entry number PRJNA943133. Data has been uploaded to the website https://dataview.ncbi.nlm.nih.gov/object/SAMN33709314.

## Abbreviations

OTU: Operational taxonomie unit
IFN-γ: Interferon- γ
IL-17: Interleukin-17
IL-1β: Interleukin-1β
SCC: Somatic cell count
EP tubes: Eppendorf tubes
PCoA: Principal Co-ordinates Analysis
SE: Standard Error
QC: Quality Control
LEfSe (LDA Effect Size): Least discriminant analysis effect size
CM: Clinical Mastitis
IgA: Immunoglobulin A
IL-12: Interleukin-12
IL-13: Interleukin-13
